# Correlation of CTGF gene promoter methylation with CTGF expression in type 2 diabetes mellitus with or without nephropathy

**DOI:** 10.3892/mmr.2014.2067

**Published:** 2014-03-24

**Authors:** HAO ZHANG, XU CAI, BIN YI, JING HUANG, JIANWEN WANG, JIAN SUN

**Affiliations:** 1 Department of Nephrology, The Third Xiangya Hospital, Central South University, Changsha, Hunan 410013, P.R. China; 2 Department of Nephrology, The Second People’s Hospital of Guangdong Province, Guangzhou, Guangdong 510317, P.R. China

**Keywords:** connective tissue growth factor, DNA methylation, diabetic nephropathy

## Abstract

Increasing evidence shows that DNA methylation is involved in the development and progression of diabetes mellitus (DM) and its complications. Previous studies conducted by our group have indicated that high glucose levels may induce the demethylation process of the connective tissue growth factor (CTGF) gene promoter and increase the expression of CTGF in human glomerular mesangial cells. Based on these findings, the aim of the present study was to investigate the methylation level of genomic DNA and the CTGF promoter in patients with type 2 DM and to analyze its possible correlation with CTGF expression. Methylation levels of the whole genomic DNA were detected by high-performance liquid chromatography in a non-diabetes control (NDM) group (n=29), a diabetes without nephropathy (NDN) group (n=37) and a diabetes with nephropathy (DN) group (n=38). CTGF promoter methylation levels were detected by methylation-specific polymerase chain reaction and bisulfite sequencing. The levels of serum CTGF were assessed using the enzyme-linked immunosorbent assay. The methylation levels of the whole genomic DNA were not significantly different among the three groups. However, the CTGF methylation levels in the two diabetes groups were significantly lower than those in the NDM group (P<0.05), with the lowest methylation level in the DN group (P<0.05). The CTGF protein levels in the DN group were significantly higher than those in the NDM and NDN groups (P<0.05). Levels of CTGF were negatively correlated with the estimated glomerular filtration rate (eGFR) and the methylation level of the promoter, while they were positively correlated with age, urinary albumin-to-creatinine ratio (UACR), blood urea nitrogen, creatinine, fasting blood sugar and postprandial blood glucose. Multiple stepwise regression analysis showed that CTGF expression was associated with the UACR, CTGF methylation level and eGFR. DNA methylation is a regulatory mechanism of CTGF expression, which is decreased in patients with DM, particularly in those with DN, and may contribute to the pathogenesis of nephropathy.

## Introduction

Diabetic nephropathy (DN) is one of the most serious microvascular complications in patients with diabetes mellitus (DM), accounting for a high percentage of the mortality and disability associated with DM (1). Despite certain drugs, including angiotensin-converting enzyme inhibitors and angiotensin receptor blockers, which can effectively reduce albuminuria and slow the progression of DN (2–4), the incidence of DN has continued to increase at a high rate. The existence of a metabolic memory in patients with DM, in which an incidence of high blood glucose increases the risk of DM-associated diseases regardless of its long-term resolution, also affects DN (5). Thus, it has been hypothesized that epigenetic mechanisms may have a role in the pathogenesis of DM, possibly promoting genetic tendencies for DM-associated complications (6). Maier and Olek (7) demonstrated that DM is regulated by DNA methylation, while Bell *et al* (8) analyzed the promoter regions of 14,495 genes in a case-control study of 192 patients with DM and found that there were 19 CpG islands associated with DN. However, there was not sufficient evidence for those differences being a cause of DN (8).

Connective tissue growth factor (CTGF) is an important cytokine involved in the development of DN, and has been shown to be closely associated with the occurrence and prognosis of DN (9,10). It has been confirmed that CTGF gene expression is sensitive to DNA methylation (11,12). The methylation state of the CTGF promoter has been shown to be negatively correlated with CTGF expression in ovarian cancer cells, and epigenetic inactivation caused by hypermethylation of the CTGF promoter plays a role in ovarian tumorigenesis (11). Chiba *et al* (12) reported abnormal CTGF gene methylation in a variety of liver cancer cell lines and primary liver cancer tissues, and suggested that CTGF methylation may be involved in liver tumorigenesis.

A previous study by our group investigated the effects of high glucose levels and 5-aza-2′-deoxycytidine (5-aza-dCyd), a selective DNA methyltransferase inhibitor, on CTGF expression and methylation levels in human glomerular mesangial cells (HMCs) (13). The study revealed that high glucose levels and 5-aza-dCyd were able to induce the demethylation process of the CTGF gene promoter and increase the expression of CTGF mRNA and protein, indicating that DNA methylation is involved in the regulation of CTGF gene expression in HMCs. However, the methylation state of the CTGF promoter has yet to be investigated in patients with DN and it remains to be elucidated whether this particular gene-specific modification is involved in the pathogenesis of DN.

The present study was designed to use high-performance liquid chromatography (HPLC), methylation-specific polymerase chain reaction (MSP) and bisulfite sequencing (BS) to investigate the methylation levels of the whole genome and the CTGF promoter in patients with type 2 DM, and to analyze the possible correlation with serum CTGF expression and the pathogenesis of nephropathy.

## Materials and methods

### Subjects

A total of 75 patients [estimated glomerular filtration rate (eGFR) ≥30 ml/min/1.73 m^2^], who had been diagnosed with type 2 DM according to the 1999 World Health Organization revised criteria for diagnosis and classification of diabetes between December 2008 and December 2009 (14) in the Department of Endocrinology and Nephrology (The Third Xiangya Hospital, Central South University, Changsha, China), were enrolled in the present study. Patients with chronic nephritis, urinary tract infection, primary hypertension and heart failure were excluded from the study. According to the Kidney Disease Outcomes Quality Initiative Clinical Practice Guidelines and Clinical Practice Recommendations for Diabetes (15) and the study by Nichols *et al* (16), these patients were divided into two groups based on their urinary albumin-to-creatinine ratio (UACR): A non-diabetic nephropathy group (NDN group; UACR <30 μg/mg, n=37) and a diabetic nephropathy group (DN group; UACR >30<300 μg/mg, n=38). All enrolled patients with DN were characterized as having diabetic retinopathy by examination of the fundus of the eye. Twenty-nine healthy volunteers, who had attended routine physical examinations at the Third Xiangya Hospital, were recruited for the control group (NDM group). The present study was designed in accordance with the Declaration of Helsinki and was performed with approval from the Ethics Committee of the Third Xiangya Hospital. Written informed consent was obtained from all participants.

### Biochemical parameter detection

Blood samples were collected after an 8-h overnight fast and 2 h after breakfast, respectively. Fasting blood sugar (FBS), postprandial blood sugar (PBS), blood urea nitrogen (BUN) and creatinine (Cr) levels were assessed using an automatic biochemical analyzer (Model no. 7600, Hitachi Co., Tokyo, Japan). Levels of glycosylated hemoglobin (HbA1c) were assessed using HPLC. First-morning, midstream urine samples were collected and centrifuged, and the supernatant was used for analysis. Urine albuminuria was assessed using an immunonephelometric method (Turbox Microalbuminaria Assay kit; Orion Diagnostica Oy, Espoo, Finland) and urine Cr was assessed using a biochemical assay and automatic biochemical analyzer (Model no. 7600, Hitachi Co.). The eGFR was calculated using the abbreviated Modification of Diet in Renal Disease equation, as follows: 186.3 × (sCr/88.4)^−1.154^ × age^−0.203^ (× 0.742 if female).

### HPLC analysis of genomic DNA methylation

Genomic DNA was extracted from peripheral blood by using the Wizard Genomic DNA Purification kit (Promega Corp., Madison, WI, USA), and the purity the of DNA was assessed using a spectrophotometer. The standard working curve was generated and the degree of genomic DNA methylation was assessed using HPLC according to the manufacturer’s instructions (Agilent 1100 system; Agilent Technologies, Inc., Santa Clara, CA, USA). Levels of genomic DNA methylation were calculated from the levels of deoxidized 5-methylcytosine (5mdC) and deoxidized cytosine (dC) detected by HPLC using the following formula: [5mdC molarity/(5mdC molarity + dC molarity)] × 100%.

### MSP analysis of CTGF promoter methylation

Methylation-specific primers were designed based on the promoter sequence of CTGF, with 5′-TCGTTTCGGTCG ATAGTTTC-3′ as the forward primer and 5′-CGAAAC CCATACTAACGACG-3′ as the reverse primer. The sequences 5′-TTGTTTTGGTTGATAGTTTT-3′ and 5′-CAA AACCCATACTAACAACA-3′ were used for the forward and reverse non-methylation-specific primers, respectively. The following thermal cycling conditions were used: Initial denaturation at 95°C for 5 min; 38 cycles of denaturation at 94°C for 45 sec, annealing at 50°C for 30 sec and extension at 72°C for 30 sec; final extension at 72°C for 7 min. The 159-bp MSP product was isolated using electrophoresis in a 1.5% agarose gel and analyzed using an ultraviolet (UV) gel imaging system (ImageQuant 350; GE Healthcare Co., Little Chalfont, UK).

### BS analysis of CTGF promoter methylation

BS primers for the CTGF promoter region were designed to avoid methylated CpGs. Once the DNA sample treated with sodium bisulfite was fully sulfonated, the BS product was amplified with forward 5′-GTTGAGAGGAGATAGTTAGTG-3′ and reverse 5′-GGTTGTTAGGGAGGGATT-3′ primers. The polymerase chain reaction (PCR) thermal cycling conditions were: Initial denaturation at 95°C for 5 min; 36 cycles of denaturation at 94°C for 45 sec, annealing at 52°C for 30 sec and extension at 72°C for 30 sec; final extension at 72°C for 5 min. The 296-bp amplification product was isolated using electrophoresis in a 1.5% agarose gel and visualized under UV light. A 10-μl aliquot of the PCR product was subjected to further sequencing by the Beijing Genomics Institute (Beijing, China). The BS primer amplification products from the samples of the three groups were compared with completely sulfonated promoter target sequences using the JellyFish 1.3 data application software (Field Scientific, LCC, Lewisburg, PA, USA). The target sequence was known to contain 39 methylated cytosine-guanine (C-G) pairs, and any remaining methylated cytosines (mCs) in the sequence indicated a methylation site. The methylation level was calculated as: (mC/C-G) × 100%.

### Enzyme-linked immunosorbent assay (ELISA) for CTGF protein detection

The CTGF protein levels in the serum were assessed using a commercially available ELISA kit (Wuhan Xinqidi Biological Technology Co., Wuhan, China), according to the manufacturer’s instructions.

### Statistical analysis

Normally distributed data are presented as the mean ± standard deviation, while non-normally distributed data are presented as the median (interquartile range). The SPSS software package (version 17.0; SPSS, Inc., Chicago, IL, USA) was used for all statistical analyses. Intergroup differences were evaluated using one-way analysis of variance (ANOVA) or one-way ANOVA with post hoc tests, wherever appropriate. Correlations between different parameters were evaluated by Pearson’s correlation test and multiple stepwise regression analyses. P<0.05 was considered to indicate a statistically significant difference.

## Results

### Comparison of clinical characteristics

There were no differences in age, gender, body mass index or systolic and diastolic blood pressure among the groups. The FBS and PBS in the NDN and DN groups were significantly higher than those in the NDM group, and HbA1c levels in the DN group were higher than those in the NDN group. BUN and Cr levels in the DN group were significantly higher than those in the NDM and NDN groups, while there was no difference in the duration of diabetes between the NDN and DN groups (Table I).

### Genomic DNA methylation levels

**Figure 1:**
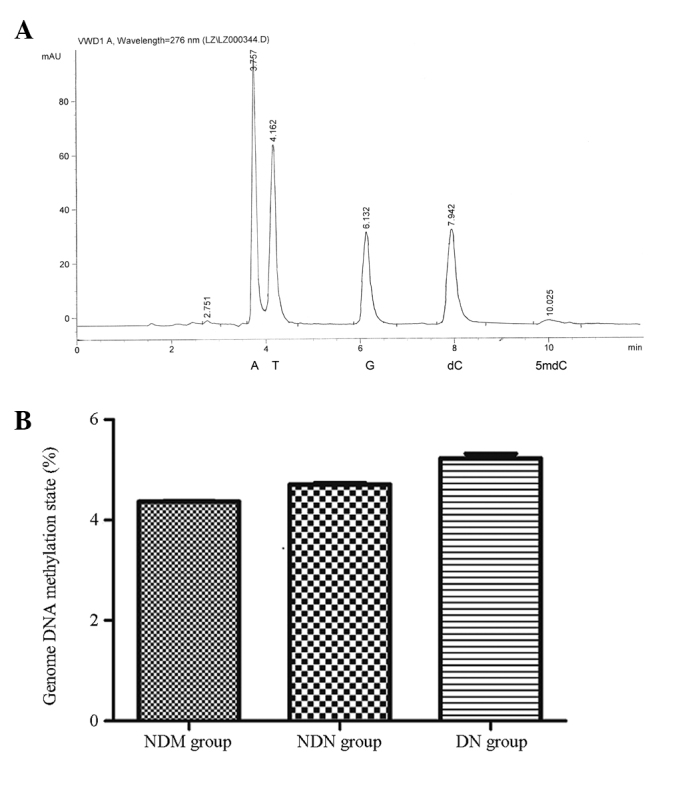
Genomic DNA methylation levels analyzed by high-performance liquid chromatography. (A) Sample chromatogram map. The peak time of sample dC was 7.942 min and that of 5mdC was 10.025 min. Nucleotides: A, adenine; T, thymine; G, guanine; dC, deoxidized cytosine; 5mdC, deoxidized 5-methyl cytosine. (B) Quantified genomic DNA methylation levels in the different groups. The genomic DNA methylation rate was 5.23±0.09% in the DN group, 4.71±0.03% in the NDN group and 4.37±0.01% in the NDM group. There were no significant differences among the groups. NDM, non-diabetes control; NDN, diabetes without nephropathy; DN, diabetes with nephropathy; VWD, variable wavelength detector.

The peak time of dC and 5mdC in the peripheral blood genomic DNA samples of the patients was 7.942 and 10.025 min, respectively, which was consistent with the standard. There was no notable peak interference due to impurities. The corresponding concentration was determined via the sample peak area (Fig. 1A). The genomic DNA methylation level, calculated using the formula [5mdC molarity/(5mdC molarity + dC molarity)] × 100%, was 5.23±0.09% in the DN group, 4.71±0.03% in the NDN group and 4.37±0.01% in the NDM group. There were no significant differences among the groups (P>0.05; Fig. 1B).

### CTGF gene promoter methylation states

**Figure 2:**
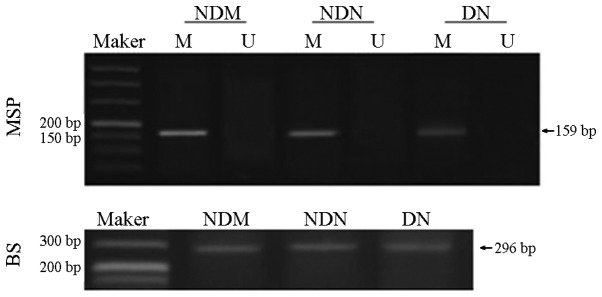
CTGF gene promoter methylation level detected by MSP. The results show a high degree of methylation in the CTGF gene promoter in the NDM group, which was moderately decreased in the NDN group and significantly reduced in the DN group. BS amplification results confirmed complete sulfonation modification of DNA in the three groups. Marker, 50 bp DNA ladder; M, amplified products with methylation-specific primers; U, amplified products with non-methylation-specific primers. CTGF, connective tissue growth factor; MSP, methylation-specific polymerase chain reaction; BS, bisulfite sequencing; NDM, non-diabetes control; NDN, diabetes without nephropathy; DN, diabetes with nephropathy.

BS amplification results confirmed complete sulfonation of the DNA in the three groups. MSP analysis revealed high levels of methylation in the CTGF gene promoter in the NDM group. Methylation levels in the CTGF promoter were moderately decreased in the NDN group (P<0.05) and significantly reduced in the DN group (P<0.01; Fig. 2). The rate of CTGF promoter methylation was 82.8% (24/29) in the NDM group, compared with 43.2% (16/37) and 31.6% (12/38) in the NDN and DN groups, respectively. Thus, the CTGF promoter in individuals in the NDM group showed significantly higher methylation levels than those in patients in the NDN or DN groups. The methylation rate in the NDM group was 1.92-fold higher than that in the NDN group, and 2.62-fold higher than that in the DN group. The methylation rate in the NDN group was 1.37-fold higher than that in the DN group.

**Figure 3:**
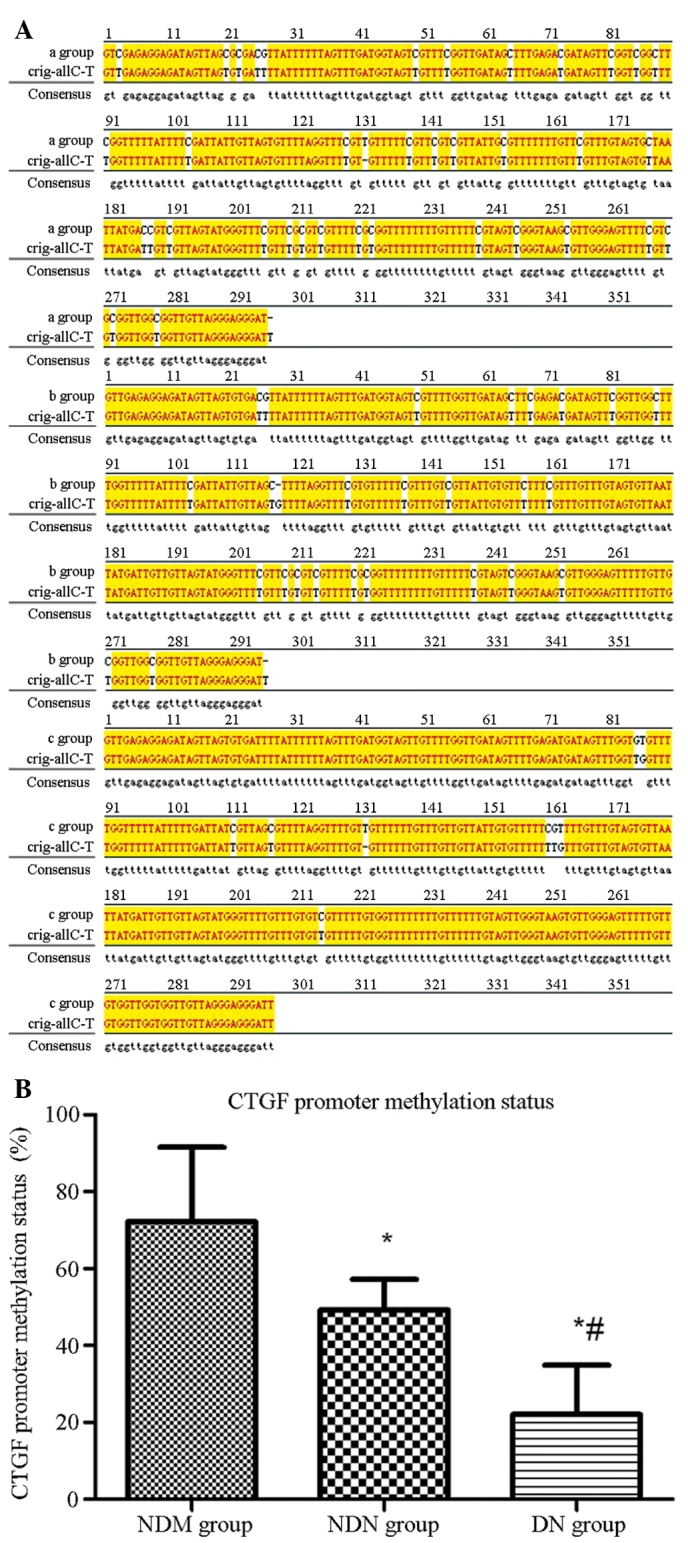
CTGF gene promoter methylation level detected by sequencing. (A) CTGF gene promoter methylation sequence mapping is shown for the (a) NDM, (b) NDN and (c) DN groups. Orig-all C-T was the original sequence fully sulfonated by sodium bisulfate. (B) Quantified CTGF gene promoter methylation levels in the different groups. The CTGF promoter methylation rate was 72.2±19.3% in the NDM group, which was significantly higher than that in the NDN group (49.2±8.0%) and the DN group (22.0±12.9%). ^*^P<0.05 versus NDM group; ^#^P<0.05 versus NDN group. A, adenine; T, thymine; G, guanine; C, cytosine; CTGF, connective tissue growth factor; NDM, non-diabetes control; NDN, diabetes without nephropathy; DN, diabetes with nephropathy.

The sequence maps for the NDM, NDN, and DN groups are shown in Fig. 3A. The target fragment was 296 bp long, and included 39 methylated C-G pairs. Orig-all C-T was the original sequence fully sulfonated by sodium bisulfate. In this sequence, all C-G pairs were sulfonated and not methylated. The base sequence difference between the samples and Orig-all C-T was compared using the JellyFish 1.3 software, and mC/C-G (39) × 100% was used to calculate the methylation degree of each sample. According to this, the calculated CTGF promoter methylation level was 72.2±19.3% in the NDM group, which was significantly higher than that in the NDN group (49.2±8.0%, P=0.045). In addition, the CTGF promoter methylation level was 22.0±12.9% in the DN group, which was significantly lower than that in the NDM group (P<0.001) and the NDN group (P=0.019) (Fig. 3B).

### Serum CTGF protein levels

**Figure 4:**
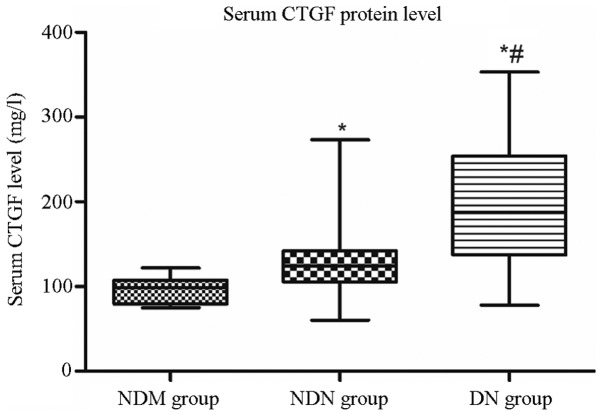
Serum CTGF protein levels were detected in different groups by ELISA. CTGF protein levels in the DN group (median, 187.55 mg/l; interquartile range, 137.33–254.08 mg/l) were significantly higher than those in the NDM group (median, 98.40 mg/l; interquartile range, 79.55–107.40 mg/l) and the NDN group (median, 124.0 mg/l; interquartile range, 105.10–142.15 mg/l). ^*^P<0.05 versus NDM group; ^#^P<0.05 versus NDN group. CTGF, connective tissue growth factor; NDM, non-diabetes control; NDN, diabetes without nephropathy; DN, diabetes with nephropathy.

As shown in Fig. 4, the CTGF protein levels in the DN group (median, 187.55 mg/l; interquartile range, 137.33–254.08 mg/l) were significantly higher than those in the NDM group (median, 98.40 mg/l; interquartile range, 79.55–107.40 mg/l; P<0.001) and the NDN group (median, 124.0 mg/l; interquartile range, 105.10–142.15 mg/l; P<0.001). In addition, the CTGF protein levels in the NDN group were significantly higher than those in the NDM group (P<0.001).

### Variables affecting CTGF expression

In order to exclude the confounding variables affecting the serum CTGF levels in patients with type 2 DM and DN, Pearson’s correlation analysis was used, and the results showed that age (r=0.197, P<0.05), UACR (r=0.591, P<0.01), BUN (r=0.443, P<0.01), Cr (r=0.393, P<0.01), FBS (r=0.278, P<0.01) and PBS (r=0.399, P<0.01) were positively correlated with CTGF expression levels, while a significant negative correlation was detected for the eGFR (r=−0.438, P<0.01), methylation levels (r=−0.367, P<0.01) and CTGF expression levels. In a multiple stepwise regression analysis using CTGF as the dependent variable and age, UACR, BUN, Cr, FBS, PBS, eGFR and methylation levels as independent variables, only UACR (β=0.452, P<0.001), eGFR (β=−0.181, P=0.034) and methylation levels (β=−0.282, P<0.001) remained significantly associated with CTGF, indicating that they are the factors influencing CTGF expression (Table II).

## Discussion

In the present study, the methylation levels of the whole genomic DNA and the CTGF promoter, as well as serum CTGF levels in patients with type 2 DM and with or without DN, were examined. DNA methylation is the most common type of apparent genetic modification, and plays essential roles in immune system imbalance, oxidative stress, inflammation, insulin resistance and fibroblast activation. In addition, DNA methylation has been proposed to be an important mechanism in the formation of renal fibrosis and cardiovascular complications in patients with chronic kidney disease (CKD) (17,18). DNA methylation has also been suggested to have a role in DM development through the silencing of zinc finger protein genes and through the maternal low-methylation syndrome that is directly associated with neonatal transient diabetes (19,20). The present study showed that the methylation levels of whole genomic DNA in peripheral blood were higher in patients in the DN or NDN groups than those in healthy volunteers; however, there were no significant differences among these three groups. In accordance with the findings of the present study, it has been previously reported that the methylation levels of the whole genome are not significantly different among patients with stages 2–4 CKD, and are not correlated with the GFR (21). The precise reason and mechanism for this remain to be elucidated. It is possible that the methylation of the overall genome reflects the sum of various degrees of gene methylation in multiple organs, thereby limiting the feasibility of detecting disease-specific methylation profiles. In addition, other systemic disorders, oxidative stress and/or chronic inflammation may complicate the methylation profile.

Although the present study shows that there is no significant difference in the methylation levels of the whole genomic DNA between patients with type 2 DM (with or without DN) and healthy volunteers, it has been reported that certain genes that are associated with type 2 DM harbor changes in methylation levels. Kuroda *et al* (22) reported that the insulin promoter of pancreatic β cells in non-diabetic mice and humans was demethylated. Methylation of the insulin promoter is involved in the regulation of insulin expression, and is associated with the levels of HbA1c expression (23). Mesangial cells are considered to be the key target cells of the pathogenic factors of DN. High glucose levels can activate CTGF transcription and expression through transforming growth factor-β-dependent and -independent pathways, thereby increasing extracellular matrix expression and leading to glomerular sclerosis (24,25). As a result, CTGF has been recognized as a central matrix-associated candidate in DN. A previous *in vitro* study performed by our group showed that 10 μM 5-aza-dCyd led to the complete demethylation of the CTGF promoter methylation region and induced the expression of CTGF, which indicates that CTGF gene expression is regulated by the methylation status of the promoter and is negatively correlated with the degree of methylation of the CpG islands (13). The results also showed that high glucose levels were able to induce CTGF promoter demethylation, indicating that high glucose levels may be involved in the regulation of CTGF gene expression by inducing DNA methylation *in vitro*.

The present study investigated the methylation status of the CTGF promoter in patients with type 2 DM with or without DN. MSP analysis showed that methylation of the CTGF gene promoter was present in 82.9% of healthy volunteers, while it was significantly reduced in patients in the NDN (43.2%) and DN (31.6%) groups. Sequencing results further confirmed the differences in the methylation status of the CTGF promoter in the three groups. CTGF serum protein expression was higher in the DN group than that in the NDN and NDM groups and higher in the NDN group than that in the NDM group, suggesting that CTGF expression may be negatively correlated with promoter methylation levels in patients with DN. Therefore, Pearson’s correlation analysis was performed to investigate which factors affect CTGF expression. The results show that CTGF expression levels are negatively correlated with eGFR or methylation levels, while they are positively correlated with age, UACR, BUN, Cr, FBS and PBS. Multiple stepwise regression analysis demonstrated that UACR, eGFR and methylation levels are associated with CTGF expression in the blood. In a study conducted by El Mesallamy *et al*, patients with DM and microalbuminuria or macroalbuminuria were shown to have significantly elevated serum CTGF levels as compared with those in control and normoalbuminuric subjects. A positive correlation was identified between the CTGF and FBS levels as well as albumin excretion rate (AER); however, multiple stepwise regression analysis revealed that only AER (β=0.5398, P<0.01) remained significantly associated with CTGF expression (26). Similarly, urinary CTGF levels were significantly elevated in patients with microalbuminuria and macroalbuminuria, and correlated with the progression of microalbuminuria (27). These results are consistent with the results of the present study.

In conclusion, the present study has demonstrated that the CTGF gene promoter exists in a low methylation state in patients with DM and particularly in patients with DN. Furthermore, it was demonstrated that the methylation level of the CTGF promoter is an independent factor of CTGF expression. These findings indicate that DNA methylation is a regulatory mechanism of CTGF expression, possibly contributing to DN pathogenesis. Understanding the role of the demethylation of the CTGF promoter in the development of DN may lead to the identification of novel strategies and/or additional therapeutic targets for the prevention and treatment of DN.

The present study had several limitations. DN is normally diagnosed by renal biopsy; however, this was not feasible in terms of clinical practice in the present study. A fundus examination was used in this study as the screening method for patients with DN, since it has been indicated that retinopathy provides sufficient evidence for glomerulopathy in patients with type 2 DM and albuminuria (15). In addition, despite the fact that previous *in vitro* studies performed by our group indicated that methylation was involved in the regulation of CTGF expression in mesangial cells, and that the present study revealed that patients with DN had decreased methylation levels of CTGF, the proposed mechanism still requires further investigation using animal models.

## Figures and Tables

**Table I:** Comparison of clinical characteristics.

Variables	NDM group, n=29	NDN group, n=37	DN group, n=38
Age (years)	57.69±11.96	56.0±9.52	58.42±9.89
Gender, male/female (n/n)	16/13	20/17	19/19
Duration of diabetes (years)	-	2.0 (1.0–7.0)	5.0 (1.0–10.0)
BMI (kg/m^2^)	22.77 (20.26–24.37)	23.74 (21.84–24.43)	23.88 (23.51–24.62)
SBP (mmHg)	126 (118–132)	128 (118–140)	130 (120–140)
DBP (mmHg)	80 (63–81)	80 (80–85)	80 (76–89)
FBS (mmol/l)	4.90 (4.15–5.30)	6.10 (5.04–7.55)^a^	6.52 (5.98–7.40)^a^
PBS (mmol/l)	6.20 (5.50–7.00)	9.20 (8.16–10.70)^a^	9.55 (9.13–10.43)^a^
BUN (mmol/l)	5.42 (4.34–5.99)	5.21 (4.03–5.99)	6.00 (5.08–7.41)^a^,^b^
Cr (μmol/l)	57.00 (52.50–68.00)	59.00 (54.00–68.00)	68.00 (57.00–80.75)^a^
HbA1c (%)	-	7.63±1.24	8.28±1.48^b^
UACR (μg/mg)	7.30 (6.41–8.22)	23.10 (16.73–26.30)^a^	102.15 (56.88–182.13)^a^,^b^
eGFR (ml/min/1.73 m^2^)	117.14±28.19	110.97±23.55	94.47±26.58^a^,^b^

Results are expressed as either the mean ± standard deviation or median (interquartile range).

a P<0.05 versus NDM group;

b P<0.05 versus NDN group.

NDM, non-diabetes control; NDN, diabetes without nephropathy; DN, diabetes with nephropathy; BMI, body mass index; SBP, systolic blood pressure; DBP, diastolic blood pressure; FBS, fasting blood sugar; PBS, postprandial blood sugar; BUN, blood urea nitrogen; Cr, creatinine, HbA1c, glycosylated hemoglobin; UACR, urinary albumin creatinine ratio; eGFR, estimated glomerular filtration rate (according to the Modification of Diet in Renal Disease formula).

**Table II:** Summary of multiple stepwise regression analysis for variables affecting CTGF.

Variables	B	SE	Standard β	t	P-value
UACR	0.387	0.074	0.452	5.220	<0.001
eGFR	−0.420	0.195	−0.181	−2.150	0.034
CTGF methylation	−35.837	9.879	−0.282	−3.628	<0.001
FBS	2.751	3.735	0.060	0.737	0.463

CTGF, connective tissue growth factor; UACR, urinary albumin creatinine ratio; eGFR, estimated glomerular filtration rate (according to the Modification of Diet in Renal Disease formula); FBS, fasting blood sugar; SE, standard error.
